# Antiretroviral treatment outcomes and survival pattern of people living with HIV in Bauchi State, Nigeria

**DOI:** 10.1371/journal.pone.0333106

**Published:** 2025-09-29

**Authors:** Ekerette Emmanuel Udoh, Adesola Zaidat Musa, Tubosun Alex Olowolafe, Chima Orakwe Obi, Oluwole Fajemisin, Taiwo Mofadeke Jaiyeola

**Affiliations:** 1 Department of Public Health, Faculty of Basic Medical and Health Sciences, Lead City University, Ibadan, Nigeria; 2 Society for Family Health, Abuja, Nigeria; 3 Nigerian Institute of Medical Research, Lagos, Nigeria; 4 Department of Epidemiology and Medical Statistics, College of Medicine, University of Ibadan, Ibadan, Nigeria; Institute of Tropical Medicine / University of Antwerp, BELGIUM

## Abstract

**Background:**

Antiretroviral therapy (ART) has greatly improved the survival and quality of life for individuals living with HIV. However, challenges in the prevention of HIV-related mortality and poor retention of patients in ART treatment pose threats to effective ART interventions. This study investigated the incidence, prevalence of ART outcomes, and survival pattern of persons living with HIV (PLHIV) on ART treatment in Bauchi state Nigeria.

**Methods:**

A retrospective cohort study was conducted to investigate antiretroviral treatment outcomes in a sample of 5,608 HIV-positive persons from two clinics over 3 years from 1st January 2020–31st December 2022. Data was extracted from an electronic medical record (EMR) from treatment facilities, and analyzed to assess the incidence and survival function estimates for ART outcomes including interruption in treatment, lost-to-follow-up (LTFU), mortality, and viral load suppression. Patient baseline demographic characteristics, clinic, pharmacy, and laboratory data were also extracted to examine associations with ART survival outcomes. Descriptives statistics were used to summarize all variables. Meanwhile, to analyze the temporal-trend plot of incidence over the study years, the data were modeled using a Generalized Linear Model (GLM) with a Poisson distribution. Kaplan-Meier survival function plots were used to estimate the survival probability of treatment outcomes, while Cox proportional hazard was modeled to identify independent predictors of survival.

**Results:**

The incidence of treatment interruption decreased steadily over the three years from 33.33 per 100 person-years (PY) in 2020 to 27.23 per 100PY in 2022. LTFU was shown to be low, decreasing significantly from 20.37 per 100PY in 2020 to 0.69 per 100PY in 2022. Incidence of mortality showed a reducing trend and ranged from 27.78 per 100PY in 2020 to 0.81 per 100PY in 2022. The high incidence observed for viral load suppression reduced slightly for the period of observation from 98.15 per 100PY to 89.62 per 100PY. Survival curves from survival analysis showed a generally decreasing survival probability trend on ART treatment outcomes, indicating a reduced risk of events over time. Additionally, participants with viral loads less than 1000 copies/ml had significantly reduced hazards of loss to follow-up (HR = 0.14, 95% CI: 0.06–0.33, p < 0.001) and death (HR = 0.26, 95% CI: 0.10–0.64, p = 0.003) compared to those with higher viral loads. Based on nutritional status of participants, overweight participants (HR = 0.07, 95% CI: 0.01–0.36, p = 0.001), as well as those with normal BMI (HR = 0.42, 95% CI: 0.18–0.98, p = 0.004) had a significantly reduced hazard of mortality compared with underweight participants.

**Conclusion:**

This study demonstrated improvements in HIV treatment outcomes. However, health challenges which limit optimal ART outcomes require targeted interventions. The study highlights the importance of integrated care and support systems for optimal treatment and survival.

## Introduction

The global HIV/AIDS epidemic continues to disproportionately burden sub-Saharan Africa, where an estimated 67% of the global HIV burden resides as of 2021 [1]. Millions of people living with HIV (PLHIV) in the region rely on antiretroviral therapy (ART) to manage the disease, transforming it from a fatal illness to a chronic condition [2]. Nigeria, the most populous country in Africa, bears a significant portion of the global HIV/AIDS burden [3]. According to the Nigeria HIV/AIDS Indicator and Impact Survey (NAIIS), Nigeria had an estimated 1.9 million people living with HIV in 2018, with approximately 1.4 million of them receiving antiretroviral therapy (ART) [4]. While ART has significantly reduced mortality and morbidity, challenges such as treatment interruptions, lost-to-follow-up (LTFU), and adverse outcomes such as drug toxicity and complications persist, particularly in resource-limited settings [5].

Recent global efforts have focused on achieving the UNAIDS 95-95-95 targets by 2030 [6]: ensuring that 95% of people living with HIV know their status, 95% of those diagnosed receive ART, and 95% of those on ART achieve viral suppression. Significant efforts in Nigeria have improved access to HIV testing, ART, and comprehensive care services [7]. However, socioeconomic factors, healthcare access, and patient-related factors remain important determinants of ART outcomes. ART outcomes, including retention in treatment, mortality, and viral load suppression, are vital to the success of HIV treatment programs. Staying in care is crucial for patients to adhere to ART and achieve viral suppression, which helps prevent HIV transmission and improves health. Studies show that consistent follow-up is linked to better viral suppression and lower death rates, while dropping out of care raises the risk of treatment failure and mortality [5]. Systems like patient tracking and community health worker involvement have helped reduce loss to follow-up, which improves retention and lowers death rates. Tackling issues like stigma, transportation, and economic barriers can also boost retention and adherence, leading to better viral suppression and reduced mortality. Barriers such as poverty, food insecurity, stigma, discrimination, and transportation costs continue to impede consistent treatment adherence, as highlighted in studies from sub-Saharan Africa [8].

The interplay between HIV and comorbid conditions, such as tuberculosis (TB) and non-communicable diseases (NCDs), further complicates ART outcomes. Studies in Kenya and Uganda have shown that co-infections with TB and NCDs can lead to treatment interruptions, poorer survival, and challenges in managing HIV [9,10]. Similarly, research from Nigeria highlighted the challenges faced by PLHIV with hypertension or diabetes in relation to ART outcomes [11]. Previous studies have suggested that early ART initiation and continuous care are associated with improved survival rates [12]. However, challenges of retention in treatment, viral load suppression, and treatment complications persist, particularly among vulnerable populations such as women, the elderly, and those with coexisting conditions [13,14]. These challenges may be due to factors including financial constraints, stigma, depression, and poor patient-provider relationships [15,16].

This study aims to provide current evidence to address the contextual issues on ART programming in the study location by investigating the incidence, prevalence, and survival on antiretroviral treatment outcomes among PLHIV in Bauchi State, Nigeria. By examining a cohort of patients over an extended period, we seek to identify key predictors of ART treatment adverse outcomes and assess their survival probabilities over time. Understanding these patterns is essential for optimizing ART programs and improving long-term outcomes for patients in the region and in other regions globally with similar socio-demographic characteristics.

## Methods

### Study design and settings

This is a retrospective cohort study of clients enrolled in ART in the last three (3) years from two treatment facilities in Bauchi State, Nigeria. Data for this study was extracted from two public facilities – one each from two Local Government Areas (LGAs) where the ART treatment program is implemented in the state. The study recruited persons aged 15–77 years old, who had been initiated on ART for HIV between January 1, 2020, and December, 31, 2022, with data retrieved in March 2023 to ensure at least three months of care for those starting treatment in December 2022. All clients who initiated ART before January 1, 2020, or after December 31, 2022, were excluded from the analysis. Data for the study was retrieved from the electronic record for the HIV database called Lafiya Management Information System (LAMIS). The LAMIS is an electronic medical record (EMR) storage and retrieval system for recording patient information for different medical domains including clinical, laboratory and pharmacy consultations. The system supports point of care (POC) services and retrospective data entry along the standard health facility workflow and enables healthcare providers to track patients across the continuum of care, generating data for improving clinical care.

In Bauchi state, HIV treatment programs aimed at reducing the prevalence of HIV have involved collaboration between the government and various partner organizations. One initiative is the Key Population Community HIV Service for Action and Response (KP-CARE-2), a project implemented by the Society for Family Health (SFH) under funding by the Global Fund. This project was designed to enhance HIV prevention, treatment, and care services in the state. The KP-CARE-2 Project of SFH implements a comprehensive HIV continuum of care by providing access to quality HIV prevention, treatment, and care services for key populations and other vulnerable groups. The project was launched in the State since 2020. The project operates through private, non-profit out-patient treatment facilities called one-stop-shop (OSS). These facilities while private work closely with public health institutions to provide comprehensive care.

At the point of ART initiation, clients undergo a comprehensive clinical assessment, including WHO staging, baseline laboratory investigations (e.g., CD4 count, creatinine, ALT [Alanine Aminotransferase, a liver enzyme measured to assess liver function]), screening for comorbidities such as tuberculosis and hepatitis B, and evaluation for mental health or substance use issues where feasible. Clients receive initial adherence counseling, information on ART side effects, and enrollment in community support systems where available. Additionally, nutritional assessment and support are provided, and eligible clients may be enrolled in differentiated service delivery models, such as fast-track or community ART groups.

During subsequent visits, the care package includes routine clinical evaluations, adherence support and counseling, viral load monitoring (per national guidelines), screening for opportunistic infections and non-communicable diseases, and psychosocial support. Clients are scheduled for monthly or quarterly clinic visits based on clinical stability and the model of care. Case managers or adherence counselors are often assigned to high-risk or newly initiated clients to ensure retention and adherence. Home-based care and follow-up visits are utilized to support clients lost to follow-up, with structured tracking mechanisms, including phone calls, SMS reminders, and community-based outreach. These interventions aim to strengthen continuity of care, improve retention, and enhance treatment outcomes.

Bauchi state and the selected study LGAs and facilities were purposefully selected for this study based on the need to evaluate an ongoing HIV program in the state. The two LGAs selected for this study for the purpose of confidentiality and anonymity will be referred to as LGA1 and LGA2. The decision not to disclose the LGAs in the study was based on the need to minimize the risk of exposing the communities to potential negative stigma. This could lead to negative stereotyping and discrimination against the residents in those communities. Clinic staff supported the retrieval of information from records based on the data extraction request by the researcher for all variables and records of PLHIV clients in the facility. Data for this study was retrieved by the program staff of the ART facilities and shared with the researchers in 11–12/04/2023.

**Fig 1:**
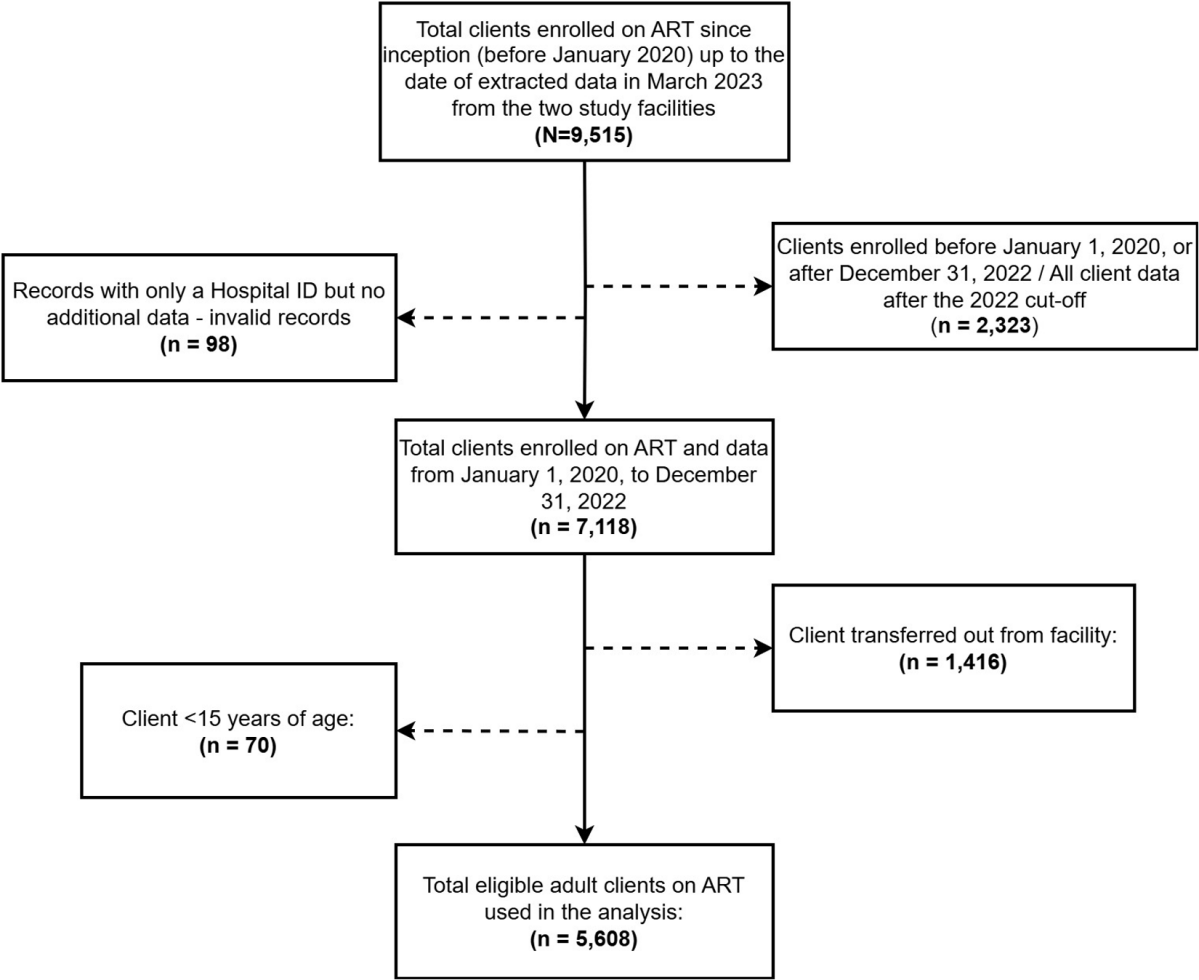
Schematic presentation of sampling of population for analysis.

Patients transferred out of the facility, cases with invalid records such as records of hospital unique identification numbers with current date of treatment status earlier than the date of registration, and cases of enrolment before or after the cut-off period were excluded in the analysis. Individuals transferred out of a facility were excluded from the study because they could potentially be transferred into another facility included in the study, leading to duplication of participants. The schema in Fig 1 below shows the recruitment process of patients for analysis. The total eligible sample for each facility was used for analysis.

### Data management and analysis

In this study, the HIV data of the clients in the facility included their clinic records, such as patient baseline data, pharmacy records for information on ART administration/regimen, and laboratory data for available information on viral load tests. CD4 counts data were not included in the analysis due to the extremely low record of data for this variable. Socio-demographic variables including age at registration, gender, educational status, occupation, marital status, and LGA of the treatment facility were extracted. Clinical related data in the extracted facility data in the study included care entry point at registration, date of ART registration, current treatment status and date of current treatment status, baseline and recurrent systolic and diastolic blood pressure reading which was used to determine the blood pressure of patients, baseline and recurrent patient weight (kg) and height (cm) which was employed to compute body mass index (BMI), baseline and recurrent functional status, baseline and recurrent clinic stage. Other variables included baseline and recurrent ART regimen, offer of prophylaxis at baseline, baseline and recurrent TB status, baseline and recurrent viral load test results. Viral load status was then categorized into three levels: “suppressed” – clients whose last VL on record is below 1,000 copies per millilitre (c/ml); “unsuppressed” – clients whose last VL record is at least 1,000 (c/ml); and “not recorded” – clients who do not have a viral load reading on record. Additionally, the date of appointments for clinic/pharmacy and laboratory visits were extracted. The entry point variable captured where clients first accessed care, including community-based organizations (CBOs), HIV testing services (HTS) centers, and other relevant service delivery points. Care entry points specified as ‘others’ included in-patient clinics, out-patient clinics, STI clinics, and Prevention of Mother-to-Child Transmission (PMTCT) services. Care entry points differ in the channels or services through which patients are initiated into ART. For instance, outreaches involve mobile or community-based efforts to identify and initiate individuals on treatment. HTS typically occur within health facilities and focuses on diagnosing individuals during routine visits. While CBO programs engage specific populations through targeted interventions and support services within the community setting. The differentiated model of care (DMOC) type variable classified clients based on their ART service delivery approach, such as multimonth dispensing (MMD), where clients receive several months’ supply of ART at once, and other models tailored to client needs and service availability. The viral load indication variable captured the reason for conducting viral load testing, including routine monitoring as part of standard care and targeted testing following Enhanced Adherence Counseling (EAC).

The main outcome variables in this study, referred to as the ART survival outcomes include: Treatment interruptions (Ever interrupted treatment) – not picking up ART drug after 28 days of appointment at any point throughout the repeat visits. LTFU – patients who did not pickup and ART drug after 28 days and never returned to take drugs. Mortality – recorded death at any point throughout treatment, and Viral load suppression – patients who achieved a viral load status of below 1,000 copies per milliliter (c/ml) at the time of censor or the end of the study duration.

To estimate the incidence of ART survival outcomes over the three years in the study, the measure of the number of new cases of ART outcome per the total observation time in a group at risk (total person-years at risk) was computed. In addition, the cumulative prevalence with their 95% CI (95% Confidence Interval, indicating the range within which the true population parameter is expected to fall with 95% confidence) of ART outcomes over the three years was also analyzed. Survival patterns of ART outcomes were investigated employing survival analysis through the Kaplan–Meier model using the *survfit* function in R software. The resulting Kaplan-Meier probability function were plotted to reveal survival patterns on ART outcomes using survival curves to visualize the step-by-step trend of the probability function of survival according to time to event. In this study censored subjects were not excluded in the study to avoid bias in the estimation of survival probabilities and the overall analysis of survival outcomes. Kaplan-Meier survival curves were also plotted for selected explanatory variables such as viral load status and BMI for each ART outcome, and the log-rank test was used to compare survival between the groups.

To identify the predictors or independent factors associated with the ART survival outcome variables, multivariate Cox proportional hazards ratios were modelled. The initial model included numerous covariates, however, the calculated Akaike Information Criterion (AIC) values were used to determine the best fit based on the model with the smallest AIC. In addition, the Cox proportional hazards assumptions for the models were tested using the Schoenfeld residuals test. Variables that did not meet the assumptions of the test were stratified in the final models. In all the analysis, missing data were excluded.

### Ethical concern

Ethical approval was sought from the Bauchi State Health Research Ethics Committee (BASHREC), with Approval No: NREC/03/11/19B/2021/018) for the implementation of the study in the state, with a waiver for consent for the extraction and utilization of patient’s data. Permission to obtain HIV patient records from treatment facilities was obtained from Society for Family Health (SFH) the organization managing the outpatient HIV treatment clinics in the two study LGAs where this study data was extracted. The data were kept strictly confidential and only deidentified data were retrieved from the facility for analysis.

## Results

From the total eligible population of 5,618 patients in the study data, the mean age of eligible patients was 36 years (SD = 8), with a 95% CI from 35 to 36. The age group distribution shows that the majority of participants fall within the age ranges of 25–49 years (87%, n = 4,880) and 20–24 years (6.2%, n = 347). Most participants were female (74%, n = 4,150) compared with males (26%, n = 1,468). The majority of respondents were married (46.9%, n = 2,472) or single (32.8%, n = 1,727). Table 1 present results on demographic characteristics of participants in the study.

**Table 1:** Demographic characteristics of study participants from two ART facilities in Bauchi state, based on data retrieved covering a three-year period from 2020 to 2022.

Characteristic	N^1^ = 5,618	95% CI^2^
**Age**	36 (8)	35, 36
**Age group**		
* 15-19*	139 (2.5%)	2.1%, 2.9%
* 20-24*	347 (6.2%)	5.6%, 6.9%
* 25-49*	4,880 (87.0%)	86%, 88%
* 50+*	244 (4.3%)	3.8%, 4.9%
**Gender**		
* *Female	4,150 (74%)	73%, 75%
* *Male	1,468 (26%)	25%, 27%
**Marital status**		
* Divorced*	472 (9.0%)	8.2%, 9.8%
* Married*	2,472 (46.9%)	46%, 48%
* Separated*	144 (2.7%)	2.3%, 3.2%
* Single*	1,727 (32.8%)	32%, 34%
* Widowed*	453 (8.6%)	7.9%, 9.4%
**Education**		
* Junior Secondary*	67 (1.4%)	1.1%, 1.8%
* None*	1,433 (29.6%)	28%, 31%
* Post Secondary*	209 (4.3%)	3.8%, 4.9%
* Primary*	1,535 (31.7%)	30%, 33%
* Quranic*	403 (8.3%)	7.6%, 9.2%
* Senior Secondary*	1,192 (24.6%)	23%, 26%
**Occupation**		
* Employed*	854 (17.1%)	16%, 18%
* Student*	140 (2.8%)	2.4%, 3.3%
* Unemployed/Retired*	3,987 (80.0%)	79%, 81%
**Study LGA**		
* LGA1*	4,100 (73%)	72%, 74%
* LGA2*	1,518 (27%)	26%, 28%

^1^Median (IQR); n (%).

^2^CI  = Confidence Interval.

The commonest care entry point into the ART treatment program was through outreaches (92.5%, n = 5,081), with other entry points being through HIV Testing Services (HTS) program (3.0%, n = 166) and community-based organization programs (CBO) (3.0%, n = 163). A majority of the patients had been on ART treatment in the facility for 18 months and above (63%, n = 3,566), with patients who had been on treatment for less than 6 months being 9.7% (n = 546), for 6–11 months being 12% (n = 693), and for 12–17 months being 14% (n = 813). At the time of registration for ART at the facility, most of the patients were HIV naive cases that had never been on ART (99%, n = 5,558), while 1% (n = 54) were cases that transferred in from a different facility into the study facility. Table 2 presents results of patients characteristics in the study.

**Table 2:** Clinic characteristics of study participants from two ART facilities in Bauchi state, based on data retrieved covering a three-year period from 2020 to 2022.

Characteristic	N^1^ = 5,618	95% CI^2^
**Care entry point**		
* CBO*	163 (3.0%)	2.5%, 3.5%
* HTS*	166 (3.0%)	2.6%, 3.5%
* Others*	82 (1.5%)	1.2%, 1.9%
* Outreach*	5,081 (92.5%)	92%, 93%
**Duration in treatment (Months)**		
* less than 6 months*	546 (9.7%)	9.0%, 11%
* 6–11 months*	693 (12%)	11%, 13%
* 12–17 months*	813 (14%)	14%, 15%
* 18 + months*	3,566 (63%)	62%, 65%
**Status at registration**		
* ART Transfer In*	54 (1.0%)	0.7%, 1.3%
* HIV Exposed Status Unknown*	6 (0.1%)	0.0%, 0.2%
* HIV + non ART*	5,558 (99%)	99%, 99%
**Blood pressure status**		
* Normal*	2,139 (40%)	38%, 41%
* Elevated*	719 (13%)	12%, 14%
* High blood pressure stage 1*	2,245 (42%)	40%, 43%
* High blood pressure stage 2*	282 (5.2%)	4.7%, 5.9%
**BMI**		
* Underweight*	416 (7.4%)	6.8%, 8.2%
* Normal*	3,800 (68%)	67%, 69%
* Overweight*	1,372 (25%)	23%, 26%
**Baseline functional status**		
* Ambulatory*	64 (1.1%)	0.8%, 1.5%
* Working*	5,513 (99%)	99%, 99%
**Baseline clinic stage**		
* Stage I*	5,534 (100%)	100%, 100%
* Stage II*	7 (0.1%)	0.1%, 0.3%
**Clinic stage at censor/end of study**		
* Stage I*	5,610 (100%)	100%, 100%
* Stage II*	3 (<0.1%)	0.0%, 0.2%
* Stage III*	1 (<0.1%)	0.0%, 0.1%
* Stage IV*	1 (<0.1%)	0.0%, 0.1%
**First ART Regimen**		
* ABC-3TC-DTG*	1 (<0.1%)	0.0%, 0.1%
* AZT-3TC-NVP*	2 (<0.1%)	0.0%, 0.1%
* TDF-3TC-DTG*	5,563 (100%)	100%, 100%
* TDF-3TC-EFV*	1 (<0.1%)	0.0%, 0.1%
**Baseline prophylaxis offered**		
* Cotrimoxazole (CTX) Prophylaxis*	248 (4.4%)	3.9%, 5.0%
* Isoniazid Preventive Therapy (IPT)*	5,277 (94%)	93%, 95%
* Not given*	93 (1.7%)	1.3%, 2.0%
**Current regimen line**		
* ART First Line Adult*	5,608 (100%)	100%, 100%
* ART First Line Children*	10 (0.2%)	0.1%, 0.3%
**Current regimen**		
* TDF-3TC-DTG*	5,608 (100%)	100%, 100%
**Baseline TB status**		
* No sign or symptoms of TB*	4,855 (88%)	87%, 89%
* Currently on INH prophylaxis*	644 (12%)	11%, 13%
* Currently on TB treatment*	2 (<0.1%)	0.0%, 0.1%
* TB positive not on TB drugs*	1 (<0.1%)	0.0%, 0.1%
**Current viral load status**		
* Greater than 1000*	98 (1.7%)	1.4%, 2.1%
* Less than 1000*	5,399 (96%)	96%, 97%
* No VL*	121 (2.2%)	1.8%, 2.6%
**Viral load indication**		
* Routine*	5,449 (99%)	99%, 99%
* Targeted – Post EAC*	47 (0.9%)	0.6%, 1.1%
**DMOC type**		
* MMD*	3,887 (100%)	100%, 100%
* Others*	2 (<0.1%)	0.0%, 0.2%

^1^Sample size ‘N’ (%).

^2^CI  = Confidence Interval: 95% Confidence Interval, indicating the range within which the true population parameter is expected to fall with 95% confidence.

**Fig 2:**
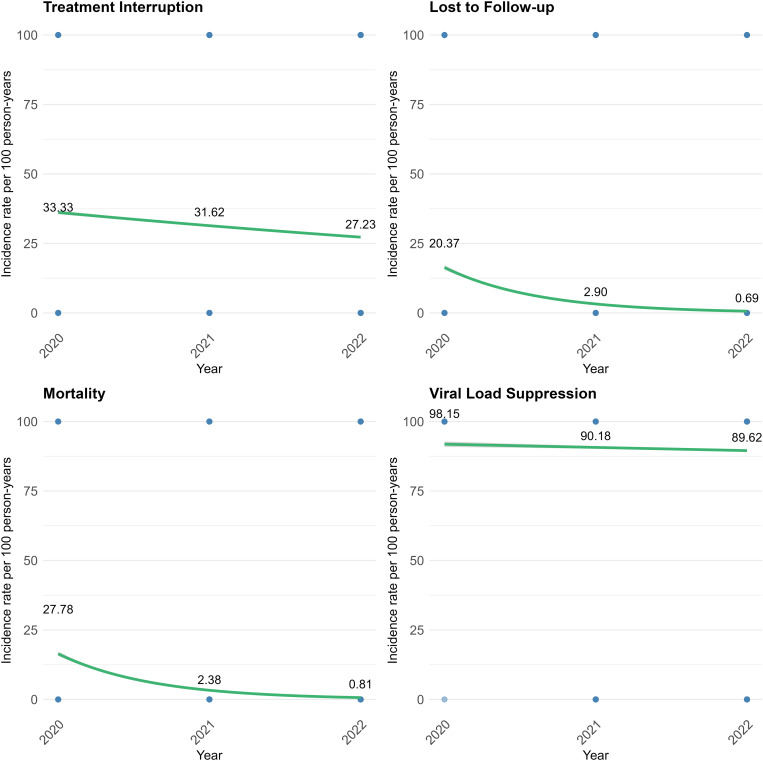
Annual incidence rate per 100 person-years trend for ART treatment outcomes.

Fig 2 presents the temporal trends in patient treatment outcomes. The plot illustrates the incidence rate per 100 person-years over successive years for the study cohort. Specifically, incidence of treatment interruption decreased steadily over the three-year period from 33.33 per 100PY in 2020 to 27.23 per 100PY in 2022. LTFU was shown to be low, decreasing significantly from 20.37 per 100PY in 2020 to 0.69 per 100PY in 2022. Incidence of mortality showed a reducing trend and ranged from 27.78 per 100PY in 2020 to 0.81 per 100PY in 2022. The high incidence observed for viral load suppression reduced slightly over the period of observation from 98.15 per 100PY to 89.62 per 100PY.

Further analysis (Table 3) showed that the cumulative prevalence for the treatment interruption was 28% (n = 1,492; 95% CI: 27–30), 1.4% (n = 79; 95 CI: 1.1–1.8) for LTFU, and mortality was 1.4% (n = 81; 95% CI: 1.2–1.8). While viral load suppression prevalence was 90% (n = 5,047; 95% CI: 89–91).

**Table 3:** Cumulative treatment outcomes prevalence rates for patients in ART treatment from year 2020 to 2022 for study participant in Bauchi State.

Characteristic	N^1^ = 5,618	95% CI^2^
**Treatment interruption**	1,592 (28%)	27%, 30%
**Lost to follow up**	79 (1.4%)	1.1%, 1.8%
**Mortality**	81 (1.4%)	1.2%, 1.8%
**Viral load** **suppression**	5,047 (90%)	89%, 91%

^1^n (%).

^2^CI  = Confidence Interval.

### Incidences of treatment outcomes according to demographic and clinical characteristics

Results in Table 4 presents incidence rates of treatment interruption and LTFU outcomes based on total person-years (PYs) according to the demographic characteristics of the participants. According to the age grouping of the participants in the study, the total PYs for treatment interruption ranged from 156 to 6341, corresponding to an incidence rate of 23.08/100 PYs (95% CI: 16.5–29.7) among participants aged 15–19 years and 21.38/100 PYs (95% CI: 20.4–22.4) among those aged 25–49 years. The age group 20–24 years exhibited a higher incidence of defaulting in treatment compared to the middle and youngest age groups. Similarly, a higher incidence of LTFU was seen among the age group 20–24 years (1.91/100 PYs, 95% CI: 0.6–3.2) with a total person-years of 418.

**Table 4:** Incidence of ART treatment interruption and LTFU by demographic characteristics among participants in HIV care facilities in Bauchi State, 2020-2022.

	Treatment Interruption				LTFU			
Characteristics	Number of Defaults	Total Person Years	Rate of Default per 100 PY (95% CI)	p-value	Number LTFU	Total Person Years	Rate of LTFU per 100 PY (95% CI)	
**Age group**								
15 - 19 years	36	156	23.08 (16.5 - 29.7)	0.011	1	156	0.64 (−0.6 - 1.9)	0.460
20 - 24 years	124	418	29.67 (25.3 - 34)		8	418	1.91 (0.6 - 3.2)	
25 - 49 years	1356	6341	21.38 (20.4 - 22.4)		67	6341	1.06 (0.8 - 1.3)	
50 + years	75	288	26.04 (21 - 31.1)		3	288	1.04 (−0.1 - 2.2)	
**Gender**								
Female	1153	5305	21.73 (20.6 - 22.8)	0.129	63	5305	1.19 (0.9 - 1.5)	0.285
Male	439	1906	23.03 (21.1 - 24.9)		16	1906	0.84 (0.4 - 1.2)	
**Marital status**								
Divorced/Separated/Widowed	324	1416	22.88 (20.7 - 25.1)	0.000	17	1416	1.2 (0.6 - 1.8)	0.215
Married	615	3030	20.3 (18.9 - 21.7)		26	3030	0.86 (0.5 - 1.2)	
Single	528	2151	24.55 (22.7 - 26.4)		28	2151	1.3 (0.8 - 1.8)	
**Education**								
None	425	1856	22.9 (21 - 24.8)	0.000	12	1856	0.65 (0.3 - 1)	0.017
Primary	392	2017	19.43 (17.7 - 21.2)		14	2017	0.69 (0.3 - 1.1)	
Quranic	73	251	29.08 (23.5 - 34.7)		1	251	0.4 (−0.4 - 1.2)	
Secondary/post-secondary	406	1851	21.93 (20 - 23.8)		26	1851	1.4 (0.9 - 1.9)	
**Occupation**								
Employed	274	1159	23.64 (21.2 - 26.1)	0.000	19	1159	1.64 (0.9 - 2.4)	0.236
Student	55	151	36.42 (28.7 - 44.1)		2	151	1.32 (−0.5 - 3.1)	
Unemployed/ Retired	1004	4862	20.59 (19.5 - 21.7)		48	4876	0.98 (0.7 - 1.3)	
**Study LGA**								
LGA1	1169	6210	18.82 (17.8 - 19.8)	0.657	72	6210	1.16 (0.9 - 1.4)	0.000
LGA2	423	1001	42.26 (39.2 - 45.3)		7	1001	0.7 (0.2 - 1.2)	

The incidence of treatment interruption was higher among males (23.03/100 PYs) compared to females (21.73/100 PYs), with total person-years of 1906 for males and 5305 for females. LTFU, however, was higher among females (1.19/100 PYs) compared to males (0.84/100 PYs), with total person-years of 5305 for females and 1906 for males, although this was not statistically significant. Treatment interruption incidence rate was highest among participants with Quaranic (29.08/100 PYs) compared to those with primary (19.43/100 PYs) or secondary education (21.93/100 PYs).

Table 5 presents incidence of mortality and viral load suppression according to participants’ demographic characteristics. The incidence of mortality was highest among the age group 25–34 years old (1.67/100 PYs) and the age group of 45 years and older (2.08/100 PYs), this was however, not statistically significant. Furthermore, the incidence of viral load suppression was highest among individuals in the youngest age group (15–24 years) and among the oldest age group (45 + years).The lowest incidence of suppressed viral load was also observed among participants with no education, employed, and student groups.

**Table 5:** Incidence of mortality and viral load suppression by demographic characteristics among participants in HIV care facilities in Bauchi State, 2020-2022.

	Mortality				Viral Load Suppression			
Characteristics	Number of Deaths	Total Person Years	Mortality Rate per 100 PY (95% CI)		Number of Suppressed Viral Load	Total Person Years	Rate of Suppressed Viral Load per 100 PY (95% CI)	p-value
**Age group**								
<15–24 years	2	156	1.28 (−0.5 - 3)	0.414	403	499	80.76 (77.3 - 84.2)	0.517
25 - 34 years	7	418	1.67 (0.4 - 2.9)		1833	2550	71.88 (70.1 - 73.6)	
35 - 44 years	66	6341	1.04 (0.8 - 1.3)		2106	2865	73.51 (71.9 - 75.1)	
45 + years	6	288	2.08 (0.4 - 3.7)		582	726	80.17 (77.3 - 83.1)	
**Gender**								
Female	52	5305	0.98 (0.7 - 1.2)	0.062	3647	4871	74.87 (73.7 - 76.1)	0.131
Male	29	1906	1.52 (1 - 2.1)		1277	1769	72.19 (70.1 - 74.3)	
**Marital status**								
Divorced/Separated/Widowed	22	1416	1.55 (0.9 - 2.2)	0.164	943	1304	72.32 (69.9 - 74.7)	0.696
Married	31	3030	1.02 (0.7 - 1.4)		2183	2814	77.58 (76 - 79.1)	
Single	23	2151	1.07 (0.6 - 1.5)		1493	1940	76.96 (75.1 - 78.8)	
**Education**								
None	23	1856	1.24 (0.7 - 1.7)	0.183	1239	1714	72.29 (70.2 - 74.4)	0.000
Primary	15	2017	0.74 (0.4 - 1.1)		1361	1856	73.33 (71.3 - 75.3)	
Quranic	9	251	3.59 (1.3 - 5.9)		360	229	157.21 (NaN – NaN)	
Secondary/post-secondary	18	1851	0.97 (0.5 - 1.4)		1287	1687	76.29 (74.3 - 78.3)	
**Occupation**								
Employed	18	1159	1.55 (0.8 - 2.3)	0.076	737	1068	69.01 (66.2 - 71.8)	0.862
Student	3	151	1.99 (−0.2 - 4.2)		115	125	92 (87.2 - 96.8)	
Unemployed/ Retired	47	4876	0.96 (0.7 - 1.2)		3510	4482	78.31 (77.1 - 79.5)	
**Study LGA**								
LGA1	63	6210	1.01 (0.8 - 1.3)	0.393	3565	5709	62.45 (61.2 - 63.7)	0.105
LGA2	18	1001	1.8 (1 - 2.6)		1359	931	145.97 (NaN – NaN)	

Note: Rates exceeding 100 occur when the number of events is high relative to the small number of person-years. This can make the rates seem exaggerated and should be interpreted with caution. The NaN values for the confidence intervals indicate that they were not computed due to the small sample sizes in some categories.

The analysis of treatment outcomes across various clinical characteristics revealed several important findings Tables 6 and 7. Treatment interruption rates varied significantly across different entry points into care, ranging from of 21.29/100 PYs (95% CI: 20.3–22.3) among participants reached through outreaches to 42.86/100 PYs among those enrolled through other entry points (Table 6). Additionally, baseline prophylaxis offered seemed to influence treatment outcomes, with patients not given prophylaxis exhibiting a higher treatment interruption rate of 38/100 PY (95% CI: 28.5–47.5), compared to those on Cotrimoxazole (CTX) Prophylaxis (23.25/100 PY, 95% CI: 19.3–27.2) or Isoniazid Preventive Therapy (IPT) (21.76/100 PY, 95% CI: 20.8–22.8). The results also showed that the incidence of treatment interruption was high among those who experienced elevated blood pressure (21.7/100 PY) and stage 1 high blood pressure (25.64/100 PY).

**Table 6:** Incidence of ART treatment interruption and LTFU by clinical characteristics *among participants in HIV care Facilities in* Bauchi State, 2020-2022.

	Treatment Interruption				LTFU			
Characteristics	Number of Defaults	Total Person Years	Rate of Default per 100 PY (95% CI)	p-value	Number of LTFU	Total Person Years	Rate of LTFU per 100 PY (95% CI)	p-value
**Care entry point**								
CBO	71	251	28.29 (22.7 - 33.9)	0.000	1	251	0.4 (−0.4 - 1.2)	0.293
HTS	66	265	24.91 (19.7 - 30.1)		5	265	1.89 (0.3 - 3.5)	
Others	36	84	42.86 (32.3 - 53.4)		1	84	1.19 (−1.1 - 3.5)	
Outreach	1380	6482	21.29 (20.3 - 22.3)		72	6482	1.11 (0.9 - 1.4)	
**Duration in treatment (months)**								
less than 6 months	37	9	411.11 (NaN – NaN)	0.000	3	9	33.33 (2.5 - 64.1)	0.000
6 - 11 months	108	95	113.68 (NaN – NaN)		25	95	26.32 (17.5 - 35.2)	
12 - 17 months	293	822	35.64 (32.4 - 38.9)		20	822	2.43 (1.4 - 3.5)	
18 + months	1154	6285	18.36 (17.4 - 19.3)		31	6285	0.49 (0.3 - 0.7)	
**Status at registration**								
ART Transfer In	24	21	114.29 (NaN – NaN)	0.009	1	21	4.76 (−4.3 - 13.9)	0.922
HIV Exposed Status Unknown	0	8	0 (0 − 0)		0	8	0 (0 − 0)	
HIV + non ART	1568	7182	21.83 (20.9 - 22.8)		78	7182	1.09 (0.8 - 1.3)	
**Blood pressure status**								
Normal	558	2990	18.66 (17.3 - 20.1)	0.022	26	2990	0.87 (0.5 - 1.2)	0.157
Elevated	217	1000	21.7 (19.1 - 24.3)		3	1000	0.3 (0 - 0.6)	
High blood pressure stage 1	671	2617	25.64 (24 - 27.3)		19	2617	0.73 (0.4 - 1.1)	
High blood pressure stage 2	75	368	20.38 (16.3 - 24.5)		1	368	0.27 (−0.3 - 0.8)	
**BMI**								
Underweight	127	411	30.9 (26.4 - 35.4)	0.326	7	411	1.7 (0.5 - 2.9)	0.456
Normal	1058	4937	21.43 (20.3 - 22.6)		39	4937	0.79 (0.5 - 1)	
Overweight	404	1842	21.93 (20 - 23.8)		14	1842	0.76 (0.4 - 1.2)	
**Baseline functional status**								
Ambulatory	18	111	16.22 (9.4 - 23.1)	–	1	111	0.9 (−0.9 - 2.7)	–
Working	1560	7039	22.16 (21.2 - 23.1)		78	7039	1.11 (0.9 - 1.4)	
**Baseline clinic stage**								
Stage I	1560	7081	22.03 (21.1 - 23)	0.100	78	7081	1.1 (0.9 - 1.3)	–
Stage II	2	10	20 (−4.8 - 44.8)		0	10	0 (0 − 0)	
Stage I	1589	7207	22.05 (21.1 - 23)		78	7207	1.08 (0.8 - 1.3)	
Stage II	0	1	0 (0 − 0)		1	1	100 (100 − 100)	
Stage III	1	1	100 (100 − 100)		0	1	0 (0 − 0)	
Stage IV	1	0	NAN (NaN – NaN)		0	0	NaN (NaN – NaN)	
**First ART regimen**								
Others	1	3	33.33 (−20 - 86.7)	1.000	0	3	0 (0 − 0)	–
TDF-3TC-DTG	1576	7137	22.08 (21.1 - 23)		78	7137	1.09 (0.8 - 1.3)	
**Baseline prophylaxis offered**								
Cotrimoxazole (CTX) Prophylaxis	103	443	23.25 (19.3 - 27.2)	0.000	5	443	1.13 (0.1 - 2.1)	0.000
Isoniazid Preventive Therapy (IPT)	1451	6668	21.76 (20.8 - 22.8)		49	6668	0.73 (0.5 - 0.9)	
Not given	38	100	38 (28.5 - 47.5)		25	100	25 (16.5 - 33.5)	
**Current ART regimen**								
TDF-3TC-DTG	1589	7197	22.08 (21.1 - 23)	–	79	7197	1.1 (0.9 - 1.3)	–
**Baseline TB status**								
No sign or symptoms of TB	1390	6697	20.76 (19.8 - 21.7)	0.242	37	6697	0.55 (0.4 - 0.7)	0.000
Currently on INH prophylaxis	171	415	41.2 (36.5 - 45.9)		18	415	4.34 (2.4 - 6.3)	
Currently on TB treatment	1	2	50 (−19.3 - 119.3)		1	2	50 (−19.3 - 119.3)	
TB positive not on TB drugs	1	1	100 (100 − 100)		0	1	0 (0 − 0)	
**Current viral load status**								
Greater than 1000	32	133	24.06 (16.8 - 31.3)	0.000	6	133	4.51 (1 - 8)	0.000
Less than 1000	1547	7017	22.05 (21.1 - 23)		38	7017	0.54 (0.4 - 0.7)	
No VL	13	61	21.31 (11 - 31.6)		35	61	57.38 (45 - 69.8)	
**viral load indication**								
Routine	1558	7105	21.93 (21 - 22.9)	0.024	34	7105	0.48 (0.3 - 0.6)	0.000
Targeted – Post EAC	21	45	46.67 (32.1 - 61.2)		10	45	22.22 (10.1 - 34.4)	

Note: Rates exceeding 100 occur when the number of events is high relative to the small number of person-years. This can make the rates seem exaggerated and should be interpreted with caution. The NaN values for the confidence intervals indicate that they were not computed due to the small sample sizes in some categories.

**Table 7:** Incidence of mortality and viral load suppression by clinical characteristics among participants in HIV care facilities in Bauchi State, 2020-2022.

	Mortality				Viral Load Suppression			
Characteristics	Number of Deaths	Total Person Years	Mortality Rate per 100 PY (95% CI)	p-value	Number of Suppressed Viral Load	Total Person Years	Rate of Suppressed Viral Load per 100 PY (95% CI)	p-value
**Care entry point**								
CBO	4	251	1.59 (0 - 3.1)	0.259	135	251	53.78 (47.6 - 59.9)	0.021
HTS	5	265	1.89 (0.3 - 3.5)		146	265	55.09 (49.1 - 61.1)	
Others	1	84	1.19 (−1.1 - 3.5)		74	84	88.1 (81.2 - 95)	
Outreach	71	6482	1.1 (0.8 - 1.4)		4577	6482	70.61 (69.5 - 71.7)	
**Duration in treatment (months)**								
less than 6 months	27	9	300 (NaN – NaN)	0.000	529	9	5877.78 (NaN – NaN)	0.000
6 - 11 months	14	95	14.74 (7.6 - 21.9)		662	95	696.84 (NaN – NaN)	
12 - 17 months	21	822	2.55 (1.5 - 3.6)		732	822	89.05 (86.9 - 91.2)	
18 + months	19	6285	0.3 (0.2 - 0.4)		3124	6285	49.71 (48.5 - 50.9)	
**Status at registration**								
ART Transfer In	0	21	0 (0 − 0)	0.642	50	21	238.1 (NaN – NaN)	0.655
HIV Exposed Status Unknown	0	8	0 (0 − 0)		4	8	50 (15.4 - 84.6)	
HIV + non ART	81	7182	1.13 (0.9 - 1.4)		4993	7182	69.52 (68.5 - 70.6)	
**Blood pressure status**								
Normal	29	2990	0.97 (0.6 - 1.3)	0.068	1903	2990	63.65 (61.9 - 65.4)	0.390
Elevated	4	1000	0.4 (0 - 0.8)		651	1000	65.1 (62.1 - 68.1)	
High blood pressure stage 1	15	2617	0.57 (0.3 - 0.9)		2030	2617	77.57 (76 - 79.2)	
High blood pressure stage 2	2	368	0.54 (−0.2 - 1.3)		253	368	68.75 (64 - 73.5)	
**BMI**								
Underweight	11	411	2.68 (1.1 - 4.2)	0.003	371	411	90.27 (87.4 - 93.1)	0.903
Normal	52	4937	1.05 (0.8 - 1.3)		3413	4937	69.13 (67.8 - 70.4)	
Overweight	8	1842	0.43 (0.1 - 0.7)		1234	1842	66.99 (64.8 - 69.1)	
**Baseline functional status**								
Ambulatory	1	111	0.9 (−0.9 - 2.7)	–	59	111	53.15 (43.9 - 62.4)	0.679
Working	80	7039	1.14 (0.9 - 1.4)		4952	7039	70.35 (69.3 - 71.4)	
**Baseline clinic stage**								
Stage I	79	7081	1.12 (0.9 - 1.4)	–	4971	7081	70.2 (69.1 - 71.3)	0.791
Stage II	0	10	0 (0 − 0)		7	10	70 (41.6 - 98.4)	
	**Last clinic stage**							
Stage I	76	7207	1.05 (0.8 - 1.3)	–	5040	7207	69.93 (68.9 - 71)	0.904
Stage II	1	1	100 (100 − 100)		3	1	300 (NaN – NaN)	
Stage III	1	1	100 (100 − 100)		1	1	100 (100 − 100)	
Stage IV	0	0	NaN (NaN – NaN)		1	0	Inf (NaN – NaN)	
**First ART regimen**								
Others	0	3	0 (0 − 0)	–	4	3	133.33 (NaN – NaN)	–
TDF-3TC-DTG	80	7137	1.12 (0.9 - 1.4)		4997	7137	70.02 (69 - 71.1)	
**Baseline prophylaxis offered**								
Cotrimoxazole (CTX) Prophylaxis	4	443	0.9 (0 - 1.8)	0.000	218	443	49.21 (44.6 - 53.9)	0.267
Isoniazid Preventive Therapy (IPT)	62	6668	0.93 (0.7 - 1.2)		4749	6668	71.22 (70.1 - 72.3)	
Not given	15	100	15 (8 - 22)		80	100	80 (72.2 - 87.8)	
**Current ART regimen**								
TDF-3TC-DTG	81	7197	1.13 (0.9 - 1.4)	–	5039	7197	70.02 (69 - 71.1)	–
**Baseline TB status**								
No sign or symptoms of TB	46	6697	0.69 (0.5 - 0.9)	0.000	4339	6697	64.79 (63.6 - 65.9)	0.066
Currently on INH prophylaxis	9	415	2.17 (0.8 - 3.6)		597	415	143.86 (NaN – NaN)	
Currently on TB treatment	0	2	0 (0 − 0)		2	2	100 (100 − 100)	
TB positive not on TB drugs	1	1	100 (100 − 100)		1	1	100 (100 − 100)	
**Current viral load status**								
Greater than 1000	7	133	5.26 (1.5 - 9.1)	0.000	0	133	0 (0 − 0)	0.000
Less than 1000	42	7017	0.6 (0.4 - 0.8)		4926	7017	70.2 (69.1 - 71.3)	
No VL	32	61	52.46 (39.9 - 65)		121	61	198.36 (NaN – NaN)	
**Viral load indication**								
Routine	43	7105	0.61 (0.4 - 0.8)	0.000	4908	7105	69.08 (68 - 70.2)	0.000
Targeted – Post EAC	6	45	13.33 (3.4 - 23.3)		17	45	37.78 (23.6 - 51.9)	

Note: Rates exceeding 100 occur when the number of events is high relative to the small number of person-years. This can make the rates seem exaggerated and should be interpreted with caution. The NaN values for the confidence intervals indicate that they were not computed due to the small sample sizes in some categories.

Results in Table 7 showed that patients with elevated blood pressure had a mortality rate of 0.4/100 PY (95% CI: 0–0.8), while those with high blood pressure stage 1 had a slightly higher rate of 0.54 (95% CI: −0.2–1.3). Patients with normal blood pressure exhibited a mortality rate of 0.97 (95% CI: 0.6–1). Underweight participants exhibited significantly higher incidence for mortality (2.68/100 PY, 95% CI: 1.1–4.2) compared with those with normal BMI.

### Survival function analysis for treatment outcomes

**Fig 3:**
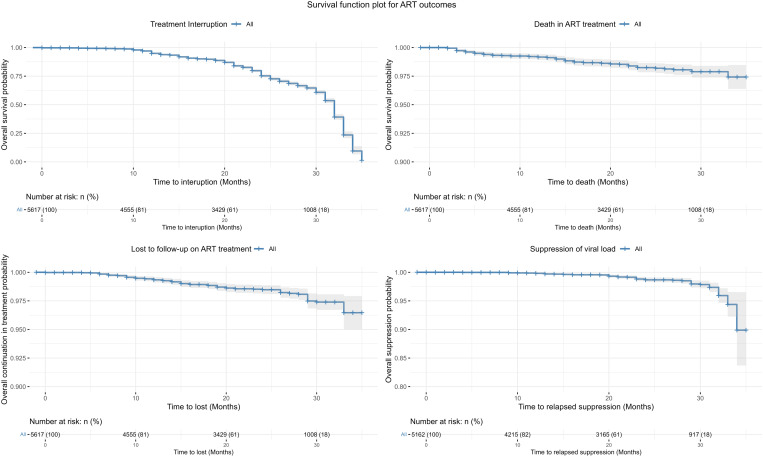
Survival curve of ART Treatment outcomes.

The survival analysis revealed a decreasing trend in the probability of treatment interruption over time. At 30 months, approximately 60% of participants had not experienced treatment interruption. Survival probabilities for LTFU, mortality, and viral load suppression showed a slight decline over time, with more than 90% of participants surviving these outcomes at 30 months. For the viral suppression survival curve, only patients with a baseline viral load below 1,000 c/ml were included. The hypothesis was that viral load would remain suppressed throughout the treatment period; otherwise, relapse would occur. Fig 3 illustrates the survival curves for these ART outcomes.

### Survival function analysis for treatment outcomes according to select clinical characteristics

**Fig 4:**
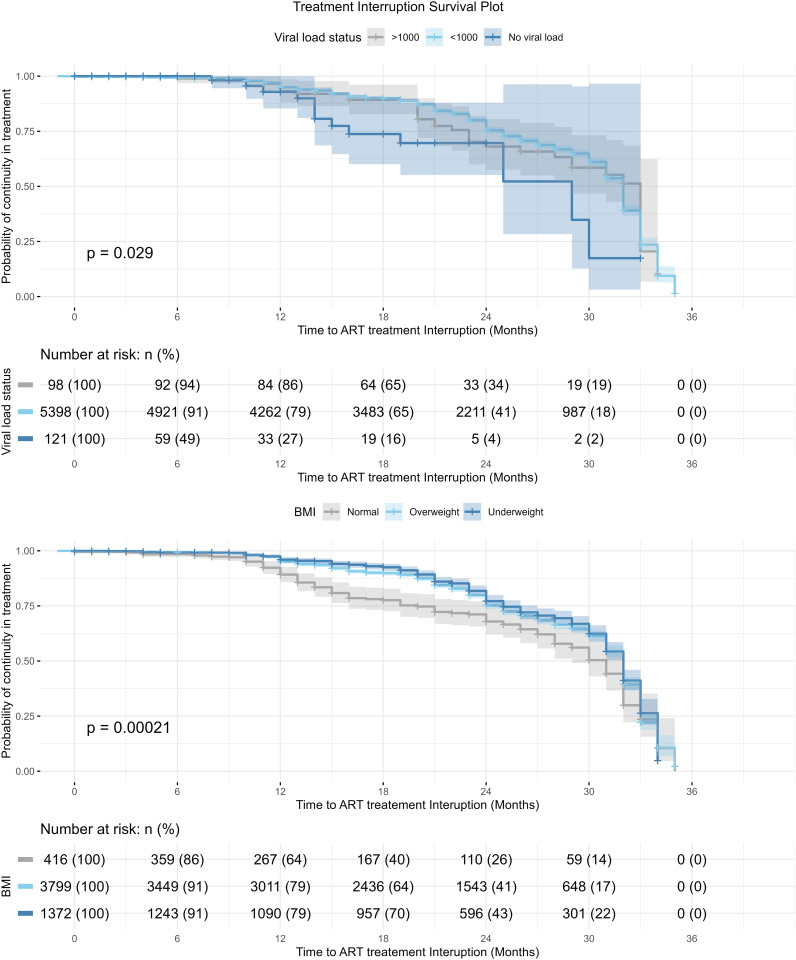
Survival function analysis of treatment Interruption for Viral load status and BMI status.

The survival curve (Fig 4) presents the survival probability for treatment interruption for the viral load status and BMI of patients. The curve indicated an increasing risk of the event over time for different viral load groupings. At 6 months, 94% with viral load > 1000c/ml and 91% with viral load < 1000c/ml were at risk, dropping to around 18% at 30 months for both groups. This survival analysis curves showed a statistically significant difference with a log-rank test p < 0.029. Based on the BMI of participants, a decline in the probability of continuing treatment (Fig 4) is exhibited. At 6 months, 91% (n = 3449) with normal BMI were at risk of treatment interruption, dropping to 17% (n = 648) at 30 months, compared to 14% (n = 59) for underweight participants at 30 months. This difference in survival estimates was statistically significant, indicating higher interruption in treatment among clients with a normal BMI over the study period.

**Fig 5:**
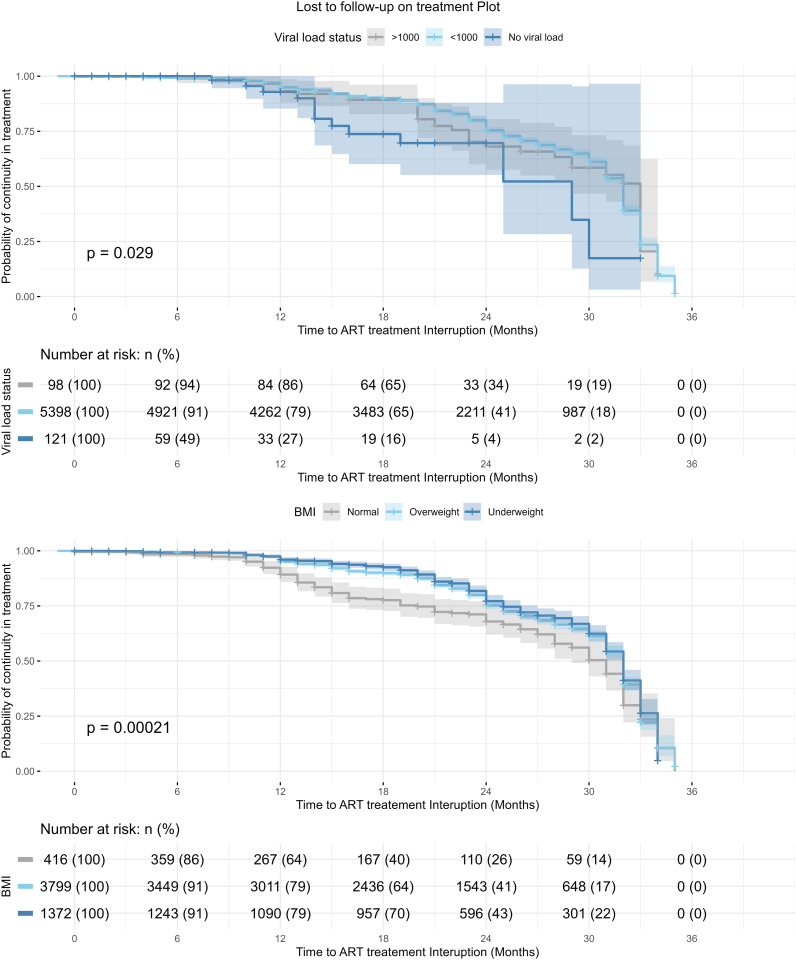
Survival function analysis for LTFU according to Viral Load status and BMI status.

Survival function analysis for LTFU based on viral load status and BMI is presented in Fig 5. The survival curve showed varying drops in the survival functions for being LTFU. At 6 months, 94% (n = 92) with both viral load status > 1000c/ml and 91% (n = 4921) with viral load status < 1000c/ml were at risk, compared to 49% (n = 59) with no viral load status reported. The risk dropped to 18% (n = 987) for viral load status < 1000c/ml and 19% (n = 19) for viral load status > 1000c/ml.

**Fig 6:**
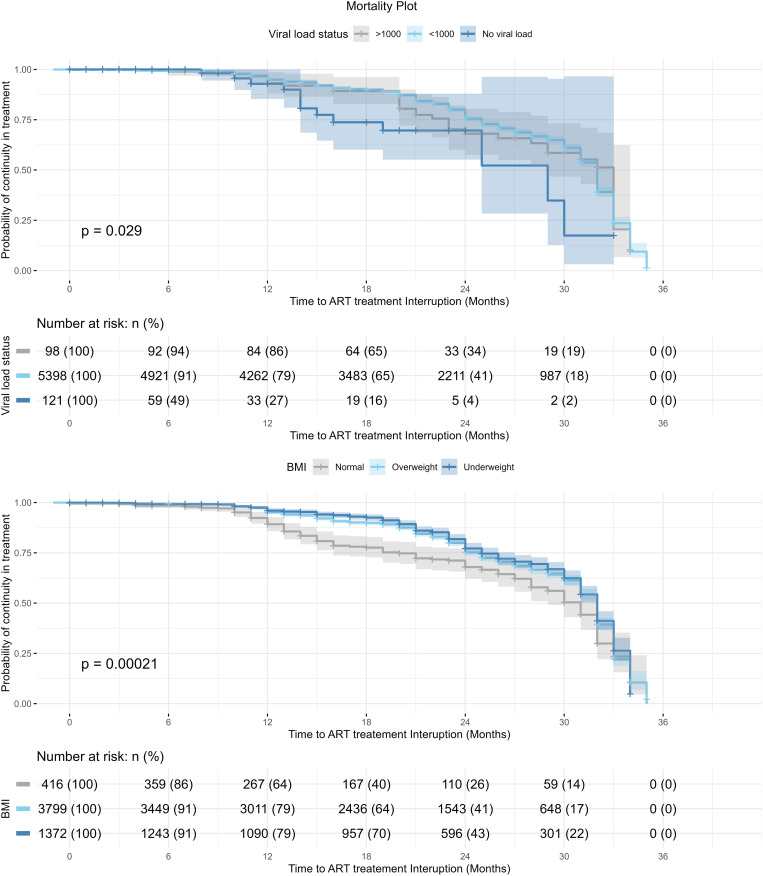
Survival function analysis of mortality for Viral load status and Treatment Interruption status.

Survival function analysis for the probability of surviving treatment without dying, based on viral load status and treatment interruption status of participants, is presented in Fig 6. For participants with a viral load status >1000 c/ml and those with no viral load status recorded, the survival curve showed a sharp decline in the probability of survival, with the risk of dying dropping from 94% (n = 92) at 6 months to 19% (n = 19) at 30 months. In contrast, for participants with a viral load status of <1000 c/ml a more gradual decrease in survival probability was shown, with the survival risk of dying decreasing from 91% (n = 4921) at 6 months to 18% at 30 months. Regarding treatment interruption status, the probability of survival decreased steadily over the study period, but the survival curves for interrupted and uninterrupted treatment were similar, showing no significant difference.

**Fig 7:**
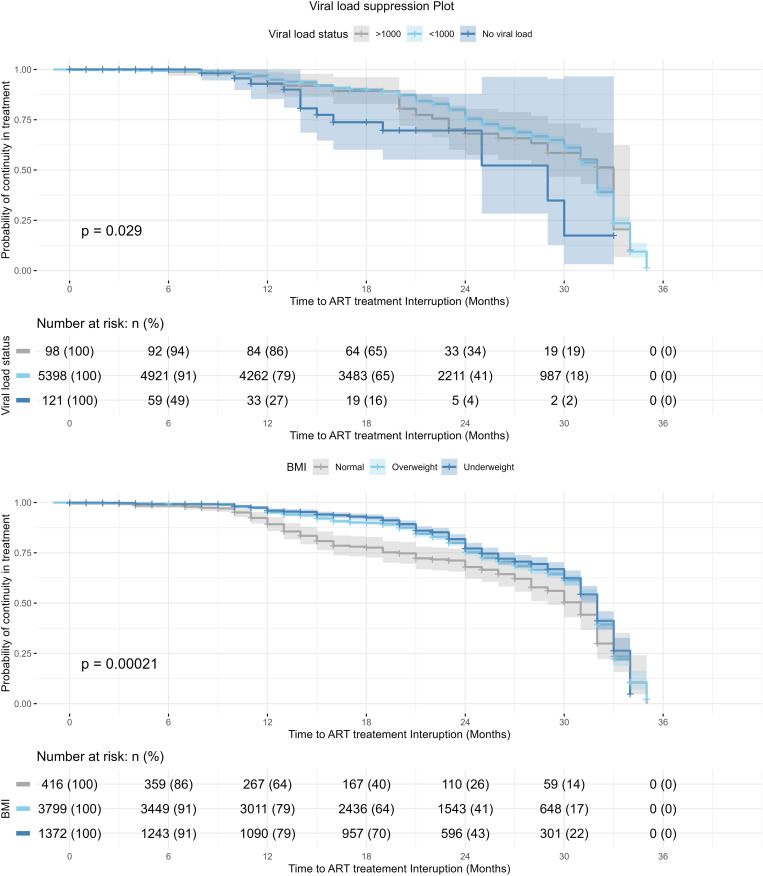
Survival function analysis of viral suppression for BMI status and Treatment Interruption status.

Survival function analysis for viral load suppression based on BMI and treatment interruption status is presented in Fig 7. Based on viral load status the survival curve showed a significantly higher probability of suppression for viral load for clients who are overweight, or have normal BMI compared to underweight participants.

### Multivariate analysis of predictors of treatment outcome

Table 8 presents the results of a multivariate Cox proportional hazards analysis examining the association between various patient characteristics and treatment outcomes, including treatment interruption, loss to follow-up, mortality, and viral load suppression. Regarding viral load suppression, participants aged 35 years and older showed a significantly lower hazard, with an HR of 0.92 (95% CI: 0.86–0.99, p = 0.020), indicating that males were 0.92 times as likely to achieve viral load suppression compared to females.

**Table 8:** Multivariate Cox proportional hazard analysis ratio of patient factors associated with treatment outcomes in HIV care, 2020-2022.

	Treatment interruption			LTFU			Death			Viral load suppression		
Characteristic	HR^1^	95% CI^1^	p-value	HR^1^	95% CI^1^	p-value	HR^1^	95% CI^1^	p-value	HR^1^	95% CI^1^	p-value
**Age group**												
* < 35 years*	—	—		—	—		—	—		—	—	
* 35 + years*	1.07	0.94 1.22	0.3	1.08	0.67, 1.75	0.7	0.60	0.31, 1.16	0.13	1.04	0.98, 1.10	0.2
**Gender**												
* Female*	—	—		—	—		—	—		—	—	
* Male*	0.84	0.74, 0.99	0.041	0.89	0.50, 1.59	0.7	1.59	0.82, 3.07	0.2	0.92	0.86, 0.99	0.020
**Marital status**												
* Divorced/Separated/Widowed*							—	—				
* Married*							0.69	0.32, 1.47	0.3			
* Single*							0.54	0.24, 1.21	0.14			
**Education**												
* None*	—	—										
* Primary*	1.33	1.14, 1.56	<0.001									
* Quranic*	1.17	0.88, 1.55	0.3									
* Secondary/post secondary*	1.42	1.25, 1.71	<0.001									
**Occupation**												
* Employed*	—	—										
* Student*	1.29	0.91, 1.75	0.2									
* Unemployed/Retired*	1.14	0.98, 1.33	0.10									
**Study LGA**												
* LGA 1*	—	—					—	—				
* LGA 2*	10.8	8.77, 13.4	<0.001				0.52	0.18, 1.53	0.2			
**Care entry point**												
* CBO*	—	—										
* HTS*	3.19	1.82, 5.60	<0.001									
* Others*	2.84	1.68, 4.82	0.001									
* Outreach*	1.77	1.34, 2.35	0.001									
**Blood pressure status**												
* Elevated:*	—	—										
* High blood pressure stage 1: 130–139/80–89*	1.02	0.84, 1.23	0.9									
* High blood pressure stage 2: 140 + /90+*	1.31	0.96, 1.78	0.091									
* Normal: 120/ < 80*	0.90	0.74, 1.09	0.3									
**Baseline offer of cotrimoxazole**												
* Not offered*				—	—		—	—		—	—	
* Offered*				0.48	0.16, 1.43	0.2	0.24	0.05, 1.11	0.067	0.14		
**Baseline offer of Isoniazid**												
* Not offered*	—	—		—	—		—	—		—		
* Offered*	0.93	0.60, 1.43	0.7	0.32	0.17, 0.60	<0.001	0.19	0.06, 0.53	0.002			
**Viral load indication**												
* Routine*	—	—					—	—		—		
* Targeted – Post EAC*	1.81	1.04, 3.16	0.035				16.3	5.59, 47.5	<0.001	18.3		
**Viral load status**												
* Greater than 1000*				—	—		—	—				
* Less than 1000*				0.14	0.06, 0.33	<0.001	0.26	0.10, 0.64	0.003			
* No VL*				11.8	4.68, 29.8	<0.001						
**BMI status**												
* Underweight*							—	—				
* Normal*							0.42	0.18, 0.98	0.044			
* Overweight*							0.07	0.01, 0.36	0.001			
**Baseline functional status**												
* Ambulatory*							—	—				
* Working*							0.64	0.09, 4.73	0.7			

^1^HR = Hazard Ratio, CI = Confidence Interval.

Male participants exhibited a significant association with treatment interruption, with an HR of 0.84 (95% CI: 0.74–0.99, p = 0.041), indicating that males were less likely to experience treatment interruption compared to females. However, there was no significant association between gender and LTFU, death, or viral load suppression. Participants with primary education exhibited significantly increased hazards of treatment interruption compared to those with no formal education, with a hazard ratio of 1.33 (95% CI: 1.14–1.56, p < 0.001). Similarly, participants with secondary/post-secondary education (HR = 1.42, 95% CI: 1.25–1.71, p < 0.001) also showed increased hazards of treatment interruption. Among occupation categories, students exhibited a higher hazard ratio for treatment interruption, with an HR of 1.29 (95% CI: 0.93–1.78, p = 0.13), compared to those who were employed – this was however, not statistically significant.

Regarding care entry points, participants entering care through HTS had a significantly higher hazard of LTFU (HR = 2.55, 95% CI: 1.47–4.43, p < 0.001) compared to those entering through CBO. Outreach as a care entry point was also associated with a higher hazard of LTFU (HR = 1.51, 95% CI: 1.15–1.99, p = 0.003).

Participants with viral loads less than 1000 copies/ml had significantly reduced hazards of LTFU (HR = 0.14, 95% CI: 0.06–0.33, p < 0.001) and death (HR = 0.26, 95% CI: 0.10–0.64, p = 0.003) compared to those with higher viral loads. Among BMI categories, overweight participants (HR = 0.07, 95% CI: 0.01–0.36, p = 0.001) and participants with normal BMI (HR = 0.42, 95% CI: 0.18–0.98, p = 0.044) had a significantly reduced hazard for mortality, compared with underweight participants..

## Discussion

This study investigated the incidence rates and the survival on antiretroviral treatment outcomes including treatment interruption, LTFU, mortality, and viral load suppression among individuals receiving HIV care over a three-year period. The findings revealed a significant reduction in treatment interruption, LTFU, and mortality rates over the study period. However, viral load suppression, while high, showed a slight decline. The results further showed that survival on treatment such as non-disruptions, retention in treatment, and not dying in treatment can be significantly influenced by patients clinical status such as having suppressed viral load and having normal BMI.

The decreased incidence of treatment interruption and LTFU shown in the results of this study indicates an improved adherence to HIV treatment and retention strategies in the treatment facilities. Although, the cumulative prevalence of treatment interruption was observed at 28%. These results are consistent with findings from another lager study in Nigeria, where prevalence of treatment interruption was reported at 32% across 16 states [17]. The significant reduction in treatment interruption may reflect the impact of enhanced patient tracking and follow-up mechanisms. The overall high prevalence can be explained by the high need for patients to consistently manage complex treatment regimens [18]. On the other hand, the cumulative prevalence of lost to follow up was 1.4%, which is relatively low compared to other studies reporting rates of 8−26% in similar settings [19,20], where barriers to consistent treatment adherence remain common despite interventions [21]. The higher incidence of mortality early in the study, with a significant drop over the study period, indicates the effectiveness of interventions and HIV care, likely due to better access to antiretroviral therapy and enhanced management of comorbidities [22]. This result corroborates findings on the decreased mortality rates after the first 12 months of ART treatment [22], and the established findings that early treatment initiation significantly impacts survival rates [23,24]. In this study the cumulative prevalence of mortality over the study period was 1.4%, which is lower compared to other studies in Nigeria that have reported mortality rates among PLHIV as high as 15% [25]. Additionally, a study in Uganda reported an in-hospital PLHIV mortality rate of 26% during the study period [26]. The higher mortality rates observed among the youngest and oldest age groups, as well as among individuals with comorbid conditions like elevated blood pressure which aligns with other studies [27,28], underscore the necessity of an integrated care approaches that will address both HIV and non-communicable disease. The cumulative prevalence of viral load suppression was 90%, which falls short of the UNAIDS 95-95-95 targets for 2030 [6]. This study demonstrated significant reductions in mortality rates and LTFU over time, indicating substantial improvements in ART outcomes. In contrast, a 2019 study in Ethiopia reported high LTFU rates (24.5%) and persistent mortality challenges due to untraceable patients, with 22.9% of traced LTFU patients deceased [29], highlighting barriers like incorrect contact information. Similarly, a 2020 study in Kenya found 27.2% LTFU and 13.5% mortality at 36 months, with factors like older age and advanced disease contributing to poorer outcomes [30]. This shows the effectiveness of the ART programme in the study sites.The results demonstrated a generally decreasing trend in survival probability among patients on ART treatment, suggesting a reduced risk of adverse events over time. The survival curve for treatment interruptions indicated a significantly higher risk in the early phases of treatment, but this risk diminished as patients continued their ART regimen. This finding is consistent with a previous study in Nigeria [17] that reported a substantial proportion of patients experiencing early treatment interruptions. Studies have also identified factors such as regimen type, facility level, and geographic region as significant contributors to the likelihood of treatment interruption, with higher risks observed in the South-West and North-East zones [17]. The trends in the survival curves for mortality and viral load suppression show a slight decrease over the study period, with only a minimal reduction in survival probability. This slight decrease in probability suggests a small increase in the risk of death and relapsed on viral load suppression. Programs should maintain effective monitoring and support strategies to ensure that even minimal risks are managed proactively, thereby sustaining long-term health outcomes and preventing potential declines in viral load suppression and mortality rates.

Several clinical and patient factors were identified as impacting treatment outcomes and survival in treatment. The findings from the adjusted Cox proportional hazard model – after controlling for other confounding factors, suggest that formal education negatively impacts on adherence to treatment as higher likelihood of treatment interruption on ART was exhibited among the population. While higher educational attainment is often linked to better health literacy, which can impact ART adherence, follow-up rates, and treatment outcomes [31,32], our findings may be explained by the nuanced realities of how formal education can introduce competing life demands. These demands may include work, schooling, and social activities, all of which can disrupt consistent access to care and adherence to treatment schedules. This finding highlights the importance of providing targeted support systems that help individuals balance educational or occupational demands with their treatment needs, thereby promoting sustained adherence and better health outcomes. [30,17] In addition, older individuals faced challenges in achieving viral load suppression. This aligns with existing literature on age-related barriers to viral load suppression [33]. Gender differences showed males had a lower hazard of treatment interruption compared to females, possibly reflecting the impact of gender-sensitive interventions. This was surprising as females are known to commonly assess health facilities for care. Higher risks were observed among those with primary or secondary education, indicating the limitation low education qualification poses to assess to ART care. This also suggests that socioeconomic factors play a role in ART care [17]. Care entry points like HTS and Outreach, which are community HIV testing initiatives, were associated with higher hazards of LTFU, emphasizing the need for stronger support systems to assist PLHIV access to healthcare in the communities in which they reside. Baseline prophylaxis with cotrimoxazole and isoniazid showed protective effects to mortality, aligning with global recommendations for integrated care strategies to improve HIV outcomes [34,35].

Despite the findings of this study, it was not without limitations. One limitation was that information on some individuals was missing due to censored observations in the retrospective data. Additionally, because many outcome events, such as LTFU, mortality, and viral load suppression, did not persist over an extended duration in the study, it was not possible to compute the mean time-to-event in our analysis. We acknowledge the potential for bias due to the characteristic that majority of participants were mainly enrolled through outreach programs. Furthermore, this study was conducted only in two LGAs from a single State in Nigeria, which may limit the generalizability of the findings to broader populations.

## Conclusion

This study demonstrates significant improvements in HIV treatment outcomes, with reduced treatment interruption, lost-to-follow-up, and mortality rates. While viral load suppression remains high, further efforts are needed to address challenges, particularly among vulnerable populations and based on individuals clinical and health challenges. The findings underscore the need for heightened integrated care, patient support systems, and adherence interventions to optimize HIV treatment and improve survival.

## References

[1] Moyo E, Moyo P, Murewanhema G, Mhango M, Chitungo I, Dzinamarira T. Key populations and Sub-Saharan Africa’s HIV response. Front Public Health. 2023;11:1079990. doi: 10.3389/fpubh.2023.1079990 37261232 PMC10229049

[2] Global HIV & AIDS statistics — Fact sheet | UNAIDS. [cited 28 Apr 2025]. Available: https://www.unaids.org/en/resources/fact-sheet

[3] Awofala AA, Ogundele OE. HIV epidemiology in Nigeria. Saudi J Biol Sci. 2018;25(4):697–703. doi: 10.1016/j.sjbs.2016.03.006 29740232 PMC5937013

[4] Federal Ministry of Health. NATIONAL GUIDELINES FOR HIV PREVENTION TREATMENT AND CARE 2020. 2020 [cited 28 Aug 2024]. Available: https://www.differentiatedservicedelivery.org/wp-content/uploads/National-guidelines-Nigeria-2020.pdf

[5] Stricker SM, Fox KA, Baggaley R, Negussie E, de Pee S, Grede N, et al. Retention in care and adherence to ART are critical elements of HIV care interventions. AIDS Behav. 2014;18 Suppl 5:S465-75. doi: 10.1007/s10461-013-0598-6 24292251

[6] Frescura L, Godfrey-Faussett P, Feizzadeh A A, El-Sadr W, Syarif O, Ghys PD, et al. Achieving the 95 95 95 targets for all: A pathway to ending AIDS. PLoS One. 2022;17(8):e0272405. doi: 10.1371/journal.pone.0272405 35925943 PMC9352102

[7] Oladele EA, Badejo OA, Obanubi C, Okechukwu EF, James E, Owhonda G, et al. Bridging the HIV treatment gap in Nigeria: examining community antiretroviral treatment models. J Int AIDS Soc. 2018;21(4):e25108. doi: 10.1002/jia2.25108 29675995 PMC5909112

[8] Buh A, Deonandan R, Gomes J, Krentel A, Oladimeji O, Yaya S. Barriers and facilitators for interventions to improve ART adherence in Sub-Saharan African countries: A systematic review and meta-analysis. PLoS One. 2023;18(11):e0295046. doi: 10.1371/journal.pone.0295046 38032918 PMC10688728

[9] Bayowa JR, Kalyango JN, Baluku JB, Katuramu R, Ssendikwanawa E, Zalwango JF, et al. Mortality rate and associated factors among patients co-infected with drug resistant tuberculosis/HIV at Mulago National Referral Hospital, Uganda, a retrospective cohort study. PLOS Glob Public Health. 2023;3(7):e0001020. doi: 10.1371/journal.pgph.0001020 37410761 PMC10325059

[10] Ngari MM, Rashid MA, Sanga D, Mathenge H, Agoro O, Mberia JK, et al. Burden of HIV and treatment outcomes among TB patients in rural Kenya: a 9-year longitudinal study. BMC Infect Dis. 2023;23(1):362. doi: 10.1186/s12879-023-08347-0 37254064 PMC10227789

[11] Akpan U, Bateganya M, Toyo O, Nwanja E, Nwangeneh C, Ogheneuzuazo O, et al. How Hypertension Rates and HIV Treatment Outcomes Compare between Older Females and Males Enrolled in an HIV Treatment Program in Southern Nigeria: A Retrospective Analysis. Trop Med Infect Dis. 2023;8(9):432. doi: 10.3390/tropicalmed8090432 37755892 PMC10536592

[12] Manosuthi W, Charoenpong L, Santiwarangkana C. A retrospective study of survival and risk factors for mortality among people living with HIV who received antiretroviral treatment in a resource-limited setting. AIDS Res Ther. 2021;18(1):71. doi: 10.1186/s12981-021-00397-1 34641922 PMC8513274

[13] Brazier E, Tymejczyk O, Wools-Kaloustian K, Jiamsakul A, Torres MTL, Lee JS, et al. Long-term HIV care outcomes under universal HIV treatment guidelines: A retrospective cohort study in 25 countries. PLoS Med. 2024;21(3):e1004367. doi: 10.1371/journal.pmed.1004367 38498589 PMC10962811

[14] Humphrey JM, Songok J, Ofner S, Musick B, Alera M, Kipchumba B, et al. Retention in care and viral suppression in the PMTCT continuum at a large referral facility in western Kenya. AIDS Behav. 2022;26(11):3494–505. doi: 10.1007/s10461-022-03666-w 35467229 PMC9550706

[15] Nutor JJ, Gyamerah AO, Alhassan RK, Duah HO, Thompson RGA, Wilson N, et al. Influence of depression and interpersonal support on adherence to antiretroviral therapy among people living with HIV. AIDS Res Ther. 2023;20(1):42. doi: 10.1186/s12981-023-00538-8 37386514 PMC10308781

[16] Ainembabazi B, Ssebunya RN, Akobye W, Mugume A, Nahirya-Ntege P, Birungi DJ, et al. Viral load suppression and retention in care among children and adolescents receiving multi-month anti-retroviral therapy refills: a program data review in Uganda. BMC Pediatr. 2024;24(1):804. doi: 10.1186/s12887-024-05295-9 39645566 PMC11624587

[17] Tomescu S, Crompton T, Adebayo J, Kinge CW, Akpan F, Rennick M. Factors associated with an interruption in treatment of people living with HIV in USAID-supported states in Nigeria: a retrospective study from 2000–2020. BMC Public Health. 2021;21:1–8. doi: 10.1186/S12889-021-12264-9 34847909 PMC8638522

[18] Villiera JB, Katsabola H, Bvumbwe M, Mhango J, Khosa J, Silverstein A, et al. Factors associated with antiretroviral therapy adherence among adolescents living with HIV in the era of isoniazid preventive therapy as part of HIV care. PLOS Glob Public Health. 2022;2(6):e0000418. doi: 10.1371/journal.pgph.0000418 36962329 PMC10022349

[19] Mushy SE, Mtisi E, Mboggo E, Mkawe S, Yahya-Malima KI, Ndega J, et al. Predictors of the observed high prevalence of loss to follow-up in ART-experienced adult PLHIV: a retrospective longitudinal cohort study in the Tanga Region, Tanzania. BMC Infect Dis. 2023;23(1):92. doi: 10.1186/s12879-023-08063-9 36788523 PMC9926646

[20] Gwynn RC, Fawzy A, Viho I, Wu Y, Abrams EJ, Nash D. Risk factors for loss to follow-up prior to ART initiation among patients enrolling in HIV care with CD4+ cell count ≥200 cells/μL in the multi-country MTCT-Plus Initiative. BMC Health Serv Res. 2015;15:247. doi: 10.1186/s12913-015-0898-9 26108273 PMC4480451

[21] Buh A, Deonandan R, Gomes J, Krentel A, Oladimeji O, Yaya S. Adherence barriers and interventions to improve ART adherence in Sub-Saharan African countries: A systematic review protocol. PLoS One. 2022;17(6):e0269252. doi: 10.1371/journal.pone.0269252 35704636 PMC9200354

[22] Ssempijja V, Namulema E, Ankunda R, Quinn TC, Cobelens F, Hoog AV, et al. Temporal trends of early mortality and its risk factors in HIV-infected adults initiating antiretroviral therapy in Uganda. EClinicalMedicine. 2020;28:100600. doi: 10.1016/j.eclinm.2020.100600 33294814 PMC7700951

[23] Anglemyer A, Rutherford GW, Easterbrook PJ, Horvath T, Vitória M, Jan M, et al. Early initiation of antiretroviral therapy in HIV-infected adults and adolescents: a systematic review. AIDS. 2014;28 Suppl 2:S105-18. doi: 10.1097/QAD.0000000000000232 24849469

[24] National Institutes of Health (NIH). Starting antiretroviral treatment early improves outcomes for HIV-infected individuals. 2015. Available: https://www.nih.gov/news-events/news-releases/starting-antiretroviral-treatment-early-improves-outcomes-hiv-infected-individuals

[25] Omololu A, Onukak A, Effiong M, Oke O, Isa SE, Habib AG. Hospitalization and mortality outcomes among adult persons living with HIV in a tertiary hospital in South-western Nigeria: A cross-sectional study. PLOS Glob Public Health. 2024;4(7):e0003487. doi: 10.1371/journal.pgph.0003487 38990938 PMC11238994

[26] Owachi D, Akatukunda P, Nanyanzi DS, Katwesigye R, Wanyina S, Muddu M, et al. Mortality and associated factors among people living with HIV admitted at a tertiary-care hospital in Uganda: a cross-sectional study. BMC Infect Dis. 2024;24(1):239. doi: 10.1186/s12879-024-09112-7 38388345 PMC10885437

[27] Haile M, Degelo T, Adilo TM, Adem FM, Gidisa B. Prevalence of Hypertension and Its Associated Factors Among Adults Living with HIV on Antiretroviral Treatment in Selected Public Hospitals in Addis Ababa, Ethiopia. HIV AIDS (Auckl). 2024;16:109–22. doi: 10.2147/HIV.S447396 38533310 PMC10963170

[28] Zhu Z, Xu Y, Wu S, Li X, Shi H, Dong X, et al. Survival and risk factors associated with mortality in people living with HIV from 2005 to 2018 in Nanjing, China. Front Public Health. 2022;10:989127. doi: 10.3389/fpubh.2022.989127 36339239 PMC9627204

[29] Assemie MA, Leshargie CT, Petrucka P. Outcomes and factors affecting mortality and successful tracing among patients lost to follow-up from antiretroviral therapy in Pawi Hospital, Northwest Ethiopia. Trop Med Health. 2019;47:52. doi: 10.1186/s41182-019-0181-6 31719791 PMC6836319

[30] Wekesa P, McLigeyo A, Owuor K, Mwangi J, Nganga E, Masamaro K. Factors associated with 36-month loss to follow-up and mortality outcomes among HIV-infected adults on antiretroviral therapy in Central Kenya. BMC Public Health. 2020;20(1):328. doi: 10.1186/s12889-020-8426-1 32171279 PMC7071670

[31] Bidzha ML, Ngepah N, Greyling T. The impact of antiretroviral treatment on the relationship between HIV/AIDS and economic growth. Economic Analysis and Policy. 2024;81:368–87. doi: 10.1016/j.eap.2023.12.005

[32] Okonkwo P, Olatoregun OJ, Abolarin O, Olajide O. Barriers to Accessing Antiretroviral Treatment Among Key Populations in Southwest Nigeria. Cureus. 2024;16(4):e59312. doi: 10.7759/cureus.59312 38817528 PMC11137604

[33] Wu G, Zhou C, Zhang X, Zhang W, Lu R, Ouyang L, et al. Higher Risks of Virologic Failure and All-Cause Deaths Among Older People Living with HIV in Chongqing, China. AIDS Res Hum Retroviruses. 2019;35(11–12):1095–102. doi: 10.1089/AID.2019.0096 31544479 PMC6862950

[34] Suthar AB, Vitoria MA, Nagata JM, Anglaret X, Mbori-Ngacha D, Sued O, et al. Co-trimoxazole prophylaxis in adults, including pregnant women, with HIV: a systematic review and meta-analysis. Lancet HIV. 2015;2(4):e137-50. doi: 10.1016/S2352-3018(15)00005-3 26424674

[35] Müller P, Velez Lapão L. Mixed methods systematic review and metasummary about barriers and facilitators for the implementation of cotrimoxazole and isoniazid-Preventive therapies for people living with HIV. PLoS One. 2022;17(3):e0251612. doi: 10.1371/journal.pone.0251612 35231047 PMC8887777

